# The impact of intra-specific diversity in the rhizobia-legume symbiosis

**DOI:** 10.1099/mic.0.001051

**Published:** 2021-04-08

**Authors:** Bryden Fields, Emma K. Moffat, Ville-Petri Friman, Ellie Harrison

**Affiliations:** ^1^​ Department of Biology, University of York, Wentworth Way, York, YO10 5DD, UK; ^2^​ Department of Animal Plant Sciences, University of Sheffield, Western Bank, Sheffield, S10 2TN, UK

**Keywords:** intraspecific diversity, mutualism, multistrain inoculant, Rhizobium leguminosarum, Rhizobia, Symbiosis

## Abstract

Rhizobia - nitrogen-fixing, root-nodulating bacteria - play a critical role in both plant ecosystems and sustainable agriculture. Rhizobia form intracellular infections within legumes roots where they produce plant accessible nitrogen from atmospheric nitrogen and thus reduce the reliance on industrial inputs. The rhizobia-legume symbiosis is often treated as a pairwise relationship between single genotypes, both in research and in the production of rhizobial inoculants. However in nature individual plants are infected by a high diversity of rhizobia symbionts. How this diversity affects productivity within the symbiosis is unclear. Here, we use a powerful statistical approach to assess the impact of diversity within the *Rhizobium leguminosarum -* clover symbiosis using a biodiversity-ecosystem function framework. Statistically, we found no significant impact of rhizobium diversity. However this relationship was weakly positive - rather than negative - indicating that there is no significant cost to increasing inoculant diversity. Productivity was influenced by the identity of the strains within an inoculant; strains with the highest individual performance showed a significant positive contribution within mixed inoculants. Overall, inoculant effectiveness was best predicted by the individual performance of the best inoculant member, and only weakly predicted by the worst performing member. Collectively, our data suggest that the *Rhizobium leguminosarum -* clover symbiosis displays a weak diversity-function relationship, but that inoculant performance can be improved through the inclusion of high performing strains. Given the wide environmental dependence of rhizobial inoculant quality, multi-strain inoculants could be highly successful as they increase the likelihood of including a strain well adapted to local conditions across different environments.

## Outline

The rhizobia-legume symbiosis is an important mutualistic association underlying the productivity of key agricultural crops [1] as well as natural ecosystems. Rhizobia convert di-nitrogen from the air into inorganic nitrogen compounds that plants can use, in exchange for nutrients and shelter in specialized root nodules. By providing the plant with nitrogen, rhizobia act as ‘biofertilisers’, reducing the need for expensive and environmentally damaging chemical fertilisers [2]. Rhizobial inoculants - where rhizobia are sewn into fields along with legume crops - thus have the potential to enable a shift to more sustainable agricultural practices. With the exception of soya cultivation, the uptake of rhizobial inoculants is generally low in legume agriculture because commercial inoculants are often ineffective or inconsistent in their ability to increase yields [3]. Rhizobial inoculants often consist of a single rhizobial genotype typically chosen for its performance with a single plant cultivar under greenhouse conditions. However, in nature rhizobia populations exhibit high genetic diversity, even across small spatial distances [4, 5]. The root systems of individual legume host plants house many dozens of root nodules, each potentially occupied by different rhizobial genotypes and in some cases multiple genotypes coinfecting single nodules [6]. Furthermore, evidence of widespread negative frequency-dependent selection of symbionts suggests that diversity may even be actively promoted by plants [7]. Rhizobial symbiont diversity is, therefore, a fundamental feature of the rhizobia-legume symbiosis that is currently unconsidered in the production of rhizobial inoculants.

The effects of symbiont genetic diversity on plant productivity is best studied in plant-mycorrhizal fungi interactions. These studies show strong productivity benefits for crop production from associations with multiple symbiont species [8, 9 , 10]. In contrast, comparatively few studies have addressed the relationship between rhizobial diversity and plant productivity. These studies have examined diversity effects over relatively small scales (<4 strains) with mixed outcomes; some studies suggest that small increases in diversity (two strains) can lead to reduced productivity [11–13], but at higher richness levels (3–4 strains) productivity is either unaffected or increased [11, 12, 14, 15]. Thus, despite the high diversity of natural rhizobial populations, it remains unclear how diversity shapes the outcome of the rhizobia-legume symbiosis particularly at high diversity levels.

Understanding how symbiont diversity contributes to the functioning and productivity of the symbiosis could be highly beneficial in the development of more successful rhizobia inoculants. This will depend both on the overall shape of this relationship – positive or negative – but also on the underlying mechanisms. Two, non-mutually exclusive mechanisms can drive positive biodiversity effects; firstly ‘complementarity’, whereby there is an inherent benefit to diversity in itself that is greater than the combined individual effect of the members of the community. This can be driven by a combination of ecological processes such as niche partitioning and facilitation. In niche partitioning community members utilise and occupy different niches and thus can exploit an environment more efficiently. Under facilitation, commensal or mutualistic interactions, such as cross feeding, allow members to be more productive in diverse communities than they would alone [16]. Where a positive biodiversity-function effect is driven by complementarity this would imply that increasing rhizobial diversity in inoculant formulations would have general beneficial effects on productivity. Under this relationship rhizobial inoculants could be improved by assembling consortia of strains which maximise phenotypic or genetic diversity.

Alternatively, greater diversity can be linked to increased productivity through ‘selection’, which posits that a community is as productive as its most productive member [16]. The selection hypothesis posits that diversity is not inherently beneficial, but diverse communities are more likely to contain individuals that are highly productive and so are, on average, more productive than less diverse communities. Where biodiversity effects are driven by selection the impact of multi-strain inoculants is more nuanced. Inoculants already aim to introduce the most productive, elite strains into the environment. Where this is achieved, increased inoculant diversity would not be expected to increase yield – as the most productive strain is already present. However, rhizobia are known to vary in productivity depending on environment and plant genotype [13, 17, 18], thus an elite strain on one farm may be a poorly performing strain on another. This makes producing truly elite inoculants – i.e. that are elite across environments – impossible. Under these conditions therefore, multi-strain inoculants could improve productivity by increasing the probability that they contain a ‘locally elite’, high performing strain for any given environment.

Distinguishing between these contrasting drivers of diversity effects is challenging. In diverse communities, ‘richness’ (the number of strains or species) cannot be independent from strain ‘identity’ (which strains/species are present). Ideally, experiments would contain all possible combinations of all community members, but such experimental designs are impractical where diversity exceeds more than a handful of members. However, powerful statistical and experimental design approaches can be used which allow variance to be partitioned between these alternative mechanisms [19] within a tractable experimental design. These methods allow the impact of community richness, which relates to complementarity, to be distinguished from the impact of member identity, which relates to selection.

In this study we address two key questions. Firstly, how does symbiont diversity alter the productivity of the rhizobia-legume symbiosis? Secondly, what mechanisms underlie this relationship? The species, *Rhizobium leguminosarum,* exhibits high genetic diversity within populations, spanning at least five distinct genospecies with divergent evolutionary histories [20]. Despite this high diversity, mobile elements are frequently shared across genospecies including symbiosis plasmids which specify plant host range [21]. Thus legume hosts, such as clover, have access to and partner with a wide diversity of *R. leguminosarum* partners. We sampled 12 high functioning *R. leguminosarum* strains isolated from white clover, *Trifolium repens,* spanning a range of diversity and phenotypic profiles. To test this we use a random partitions design following the methods of [19]. We find that increasing symbiont diversity does not measurably alter plant productivity. However we do detect an impact of strain identity within the inoculant, with certain strains contributing significantly to productivity effects when present. This supports a selection model for biodiversity-ecosystem function and suggests that increasing inoculant diversity could increase the probability of introducing a beneficial strain.

## Methods

### Strain selection and characterisation

Twelve strains of *Rhizobium leguminosarum* symbiovar *trifolii (Rlt*) were selected from a strain collection isolated previously from root nodules of white clover, *Trifolium repens* [21]. To maximise diversity, strains were selected to span three diverse genospecies [5] - A, C and E (Fig. S1, available in the online version of this article) - across a range of genetic distances.

To gain an estimate of phenotypic diversity strains were grown across a range of environmental variables. All strains were grown in modified Tryptone Yeast broth (TY) conditions (5 g Tryptone, 2.5 g Yeast Extract, 1.47 g CaCl_2_, per litre volume) in 96-well plates. TY broth was altered by modifying growth temperature (4, 10, 15, 20, 28 °C), pH at 28 °C (pH 4, 5 and 6, 6.68), and nutrient concentration at 28 °C (100, 25, 12.5, 6.25, 3.125 % TY). Strains were inoculated into 200 µl of each manipulated TY media treatments using a sterilised metal replicator (~0.2 µl, Boenik). Bacterial density (OD_600_) at 48 h was used as a proxy of rhizobial growth for all measured TY growth traits. Strains were grown in triplicate for all TY growth treatments and the average was calculated across replicates for each strain.

### Diversity-function relationship

Inoculant populations were assembled following a random partitions design [19] with five diversity levels; 1, 2, 4, 6, 12. Within an experimental random partitions set the total (12 strain) genotype pool is randomly partitioned according to each diversity level - i.e. at the four strain diversity level the 12 strains are assigned to one of three communities, at the six strain diversity level the 12 strains are assigned to one of two communities etc. Strains were partitioned using randomised calling in R and this process was repeated twice to create two experimental sets, each made up of 24 communities. All communities were repeated three times with replicates initiated for three consecutive weeks. Communities were inoculated into single white clover seedling and each replicate block contained an uninoculated control to monitor for cross contamination. Thus (24 communities × 2 blocks) +1 control × 3 replicates=147 plants in total. The resulting experimental design therefore spans both a range of genotype richness (1–12 strains), genetic diversity (average nucleotide identity (ANI) of 0.999 to 0.954) and phenotypic diversity. Phenotypic diversity was estimated as the range (min to max) of values for PC1 from a principal components analysis of phenotypic values. Across multi-strain communities this varied across two orders of magnitude (0.0124–10.192).

Single plants were grown in individual pots under anexic conditions. Seeds were sterilised in 3 % household bleach for 30 mins and washed four times in sterile water. They were germinated on sterile filter paper for 5 days at room temperature. Seedlings were transplanted into 250 g twice-autoclaved growth substrate - 5 : 4 : 1 sand:vermiculite:Levington Advance M3 compost - to mimic low nitrogen field conditions. Plants were watered with 5 ml sterile water and 1 ml N-free Jensens solution [22] prior to inoculation.

Bacterial strains were streaked from glycerol stocks onto TY agar and each replicate culture was founded by a single clone in 5 ml TY broth. These were grown for 72 h to saturation and equal volumes of each strain combined following the experimental design and mixed well. Then 200 µl inoculant culture was added to the seeding at the root-shoot interface.

Plants were placed in autoclaved ‘Sunbags’ with a sterilised watering tube topped by a 0.44 µm filter for watering. Bags were loosely sealed to allow air exchange but minimise contamination. They were grown for 40 days (until first flowering) in a greenhouse chamber at 16 h daylight, 23 °C/20 °C day/night temperatures. They were watered weekly with 1 ml Jensens solution with the volume increasing each week by 1 ml and enough water to maintain visible water content as required.

Plant productivity was measured at total plant biomass while symbiont productivity was measured as both nodule weight and number. At harvest, above ground biomass was removed, the number of leaves counted, dried at 45 °C for 48 h and weighed. Roots were gently washed and all nodules were counted and removed. The remaining roots were dried as above and weighed. Nodules were weighed while wet due to their small size.

## Analysis

To observe the phenotypic variation across strains grown in manipulated TY growth conditions, a principal components analysis (PCA) was calculated with R prcomp using singular value decomposition on log transformed, centred and scaled data. For each strain, the average bacterial density (OD_600_) at 48 h was calculated across three replicates for each TY treatment as input into the PCA. To test for the significance of the PCA clustering of strains by genospecies, phenotype data was converted to Euclidean distance and permutational analysis of variance (PERMANOVA) was calculated using adonis from the R vegan package (permutation free, 999 permutations). The significance of genospecies differences were determined with post-hoc testing using pairwise.adonis() and Bonferroni *P*-value correction.

Analysis of inoculant performance was performed in R using the nlme and lm packages. Separate models were fitted for three variables; total plant biomass (root+shoot dry weight), nodule number and nodule wet weight. Single strain effects were first analysed using linear mixed effects models (lme) with experimental block (started on weeks 1–3) as a random effect. Firstly we tested for productivity effects in single strain inoculants due to strain genetic background (divided by genospecies) and phenotype (estimates of phenotypic variance taken from PC1 of the PCA analysis). Each variable was analysed in a mixed effects model using lme() with replicate block as a random effect. We also tested for significant differences between strains. For which we ranked by average value and set the strain with the lowest value as the intercept.

Diversity effects were analysed following the statistical approach outlined by [19]. To account for random variance due to experimental block a model was initially fitted with replicate block as a main effect and residuals used in the following analysis. Dependent variables were analysed in a set of sequential linear models testing plant biomass, nodule number and weight. Variance was partitioned by factors: genotype richness (R, continuous), average ANI, strain identity, richness (R, factorial), partition group (Q) and community ID (M) [19]. The sequential model (where each variable is added to the model in order) allows for the overlap between variance attributed to genotype richness and strain identity, as these effects are, to some extent, confounded, i.e. strain identity in a given inoculum cannot be independent of the number of strains (richness) of that inoculum and vice versa. Strain identity was included in the analysis as a set of variables describing the presence or absence of each strain in each inoculant. Thus coefficients for each strain variable amount to the contribution of each strain to the productivity of each inoculant compared to the average strain effect.

## Results

### Phenotypic variation among strains

Comparison of growth responses across different abiotic stress conditions showed significant variation between strains, which could be partially explained by variation between genospecies. Strains were grown under manipulated Tryptone Yeast (TY) media differing in temperature, pH, and nutrient concentration. Principal components analysis (PCA) identified the first four principal components (PC) as showing individual percentage variances more than 5 % (66.81, 9.62, 8.05, 6.86), with a cumulative total variance of 91.33 %. The first two principal components accounted for the most variance and totalled 76.42 %. In general, high growth in the majority of TY media treatments were negatively loaded on PC1, with the exception of growth in 100 % TY at pH 4 which was positively loaded. On the other hand, the ability to grow at 4 °C in 100 % TY was strongly positively loaded on PC2. Strains were found to significantly cluster by genospecies across PC1 (Fig. S2), and PERMANOVA revealed this separation of genospecies was significant (PERMANOVA: F_2,11_=2.7975, *P*=0.045). Furthermore, post hoc testing revealed that genospecies E strains significantly differed from genospecies C (*P*=0.029, Bonferroni adjusted *P*=0.087), likely owing to their high growth densities across TY treatments compared to other strains.

### Symbiotic performance of individual inoculants

**Fig. 1.**
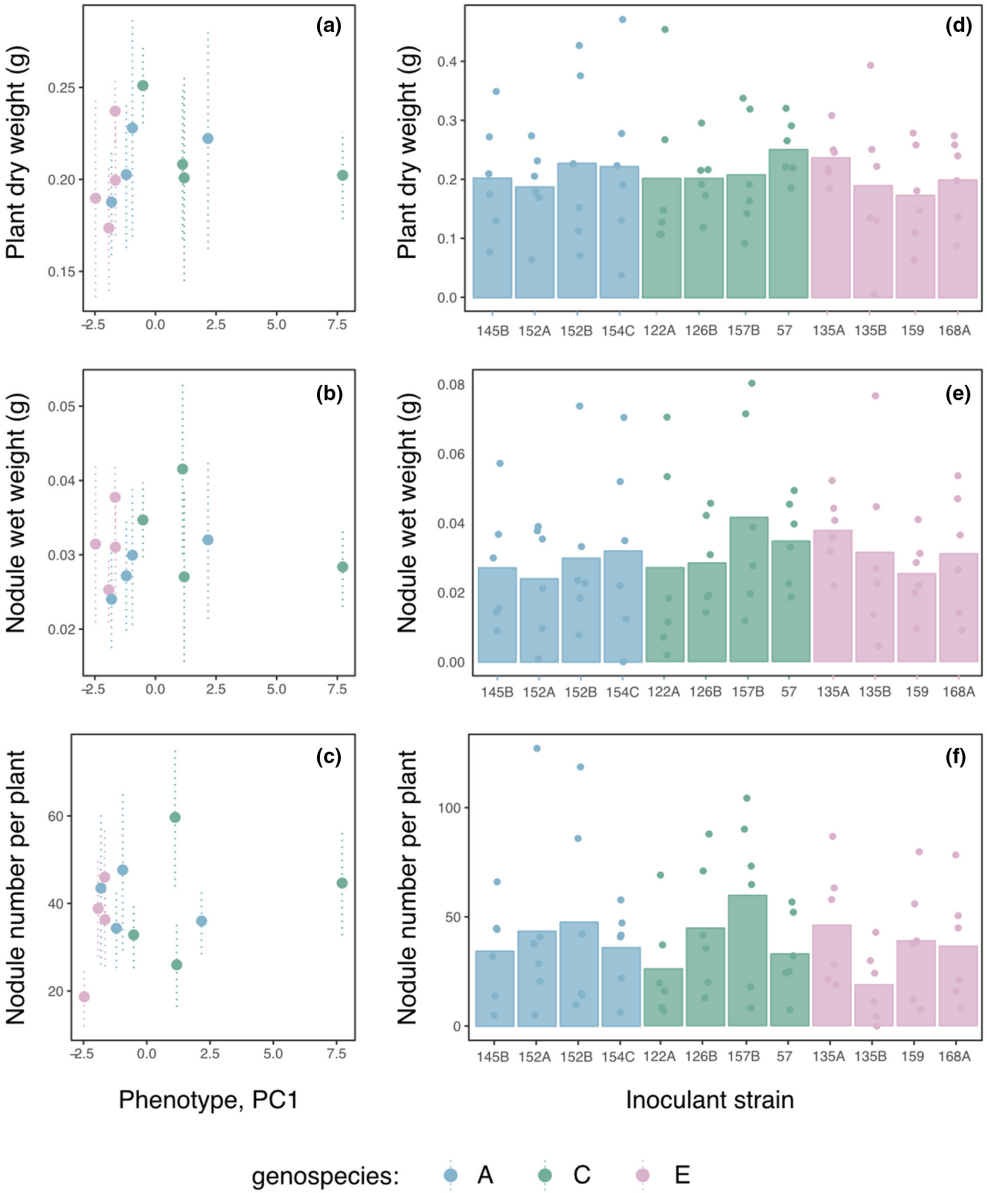
Performance of single strain inoculants. (a–c). Interaction between symbiotic productivity - as (**a**) Total plant biomass (dry weight), (**b**) nodule biomass (wet weight), (**c**) nodule number - and phenotype for each strain grown in single strain inoculants. (d–f) Mean productivity of single strain inoculants showing replicate values (filled circles) and standard error (lines). Colours denote genospecies of each strain.

All single strain inoculations successfully formed root nodules while uninoculated plants were nodule free. We detected no significant differences between individual strains in performance. Single strain isolates were examined for effects of genetic background (genospecies) and phenotype (using PC1 values of the PCA, as this contains >66 % of the variance between strains). Across the three measured variables - total plant dry weight, nodule weight and number - there was no significant effect of either genotype or phenotype on measures of productivity (*P* >0.6,) (Fig. 1a) and no interaction between them (*P* >0.25). No significant differences were observed between the performance of individual strains (*P* >0.05)(Fig. 1d, e).

### Impact of diversity on performance

The impact of genotypic and phenotypic richness on nodule formation and plant biomass was estimated in a sequential linear model (Supplementary Table 1). We quantified two metrics for rhizobia genetic diversity: genotype richness was simply a count of the number of strains in a mixture, whereas average nucleotide identity (ANI) measures the mean relatedness between strains in the inoculant. An estimate of phenotypic diversity was derived from the PCA analysis, using PC1 values as an estimate of phenotype we estimated phenotypic diversity as the range (highest minus lowest PC1 values) of strain phenotypes within an inoculant.

**Fig. 2.**
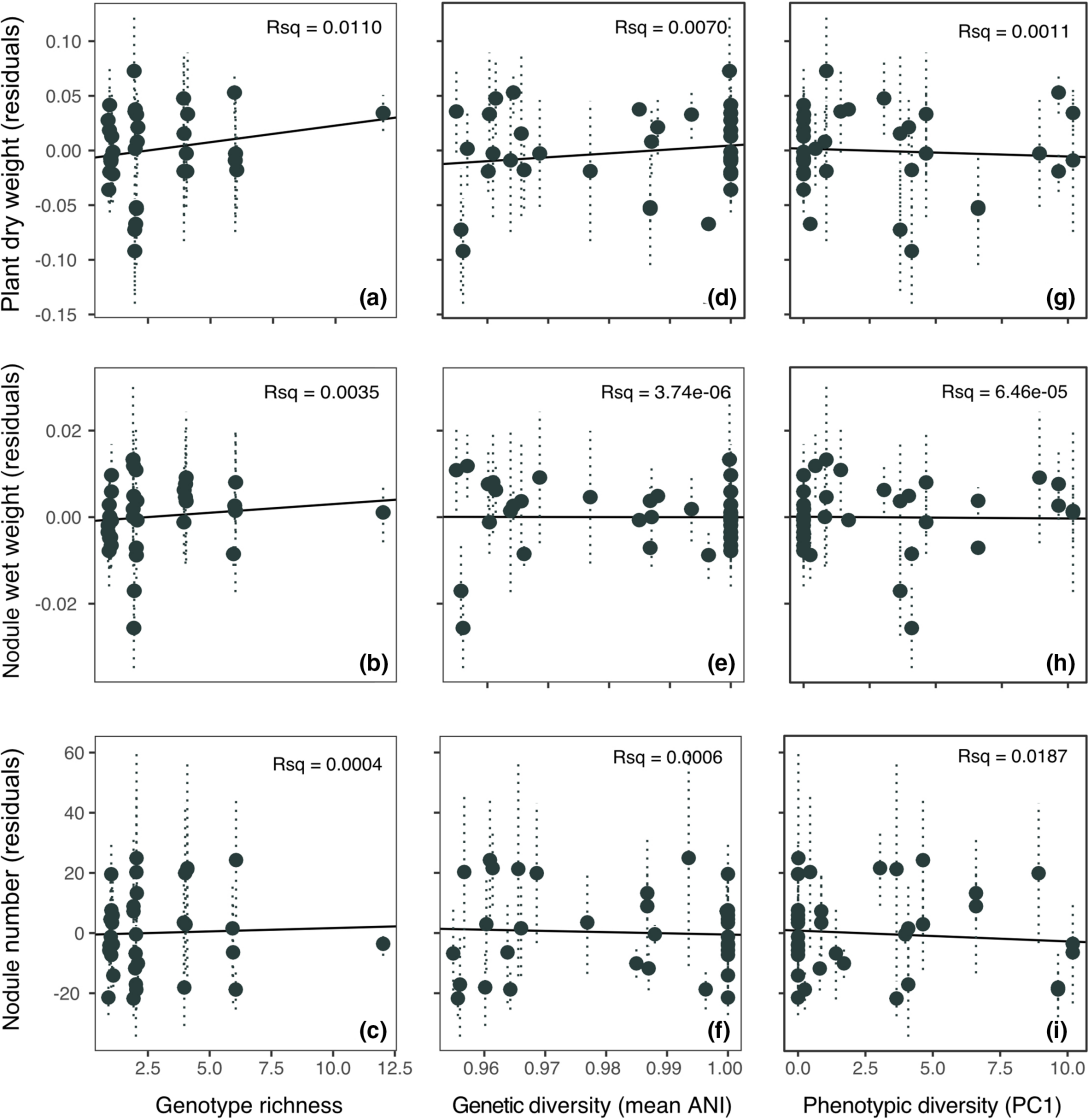
Effect of inoculant diversity on plant and rhizobial productivity. Panels show impact of diversity measured in three ways; as genotype richness (number of genotypes per inoculant, left hand panels (a–c)), genetic diversity (mean ANI, **d–f**) and phenotypic diversity (PC1, right hand panels, **g–i**). Three measures of symbiosis performance were analysed, firstly plant productivity (Total plant biomass, **g**) top panels, nodule biomass (wet weight, **g**) middle panels, and nodule number, bottom panels. Points show mean effects for each inoculant as model residuals with standard error. Residuals were plotted to remove the variance attributable to experimental block.

Measures of plasmid and symbiont productivity showed a generally weak, positive relationship with diversity, in particular genotype richness. Fig. 2 shows the interaction between each measured parameter of plant and rhizobial productivity (plant biomass, nodule weight and nodule number) across three measures of diversity (genotype richness, genetic diversity and phenotypic diversity). However in all cases these relationships were not significant (Fig. 2, *P* >0.1). A positive response was most pronounced for genotype richness (Fig. 2a, b and c) across all three measured parameters, but most clearly for plant biomass (Fig. 2a, genotype richness: Rsq=0.0109). Rhizobial investment, measured as nodule weight (Fig. 2b, genotype richness: Rsq=0.00354) or nodule number (Fig. 2c, genotype richness: Rsq=0.000391), had a shallower relationship with community diversity. Average relatedness (ANI) within the inoculants had a far weaker effect on productivity compared with genotype richness (Fig. 2). As the two diversity variables are not independent from each other the order in which they appear in the model will alter the model outcome. To check the importance of this on the relative significance of these two measures of diversity the order in which they appear in the sequential model was reversed. However, this did not materially change the outcome; in the case of plant biomass, where the largest positive response was observed, placing average relatedness first in the model actually increased the Rsq of genotype richness and decreased that of relatedness (Table S2). Estimates of phenotypic diversity within inoculants had no effect (*P* >0.6 for plant biomass, nodule weight and number). If anything phenotypic diversity had a slight negative impact on productivity. Again, this was most pronounced for plant biomass (Rsq=0.007).

Across all models, replicate block showed a strongly significant effect (*P* <0.001), suggesting that variance due to block effects was large. This likely limited the power of the experiment to determine small effect sizes. Importantly however these weak, potentially positive relationships do support the finding that increased rhizobial diversity does not reduce the effectiveness of rhizobial inoculants.

### Impact of strain identity on the performance multistrain inoculants

**Fig. 3.**
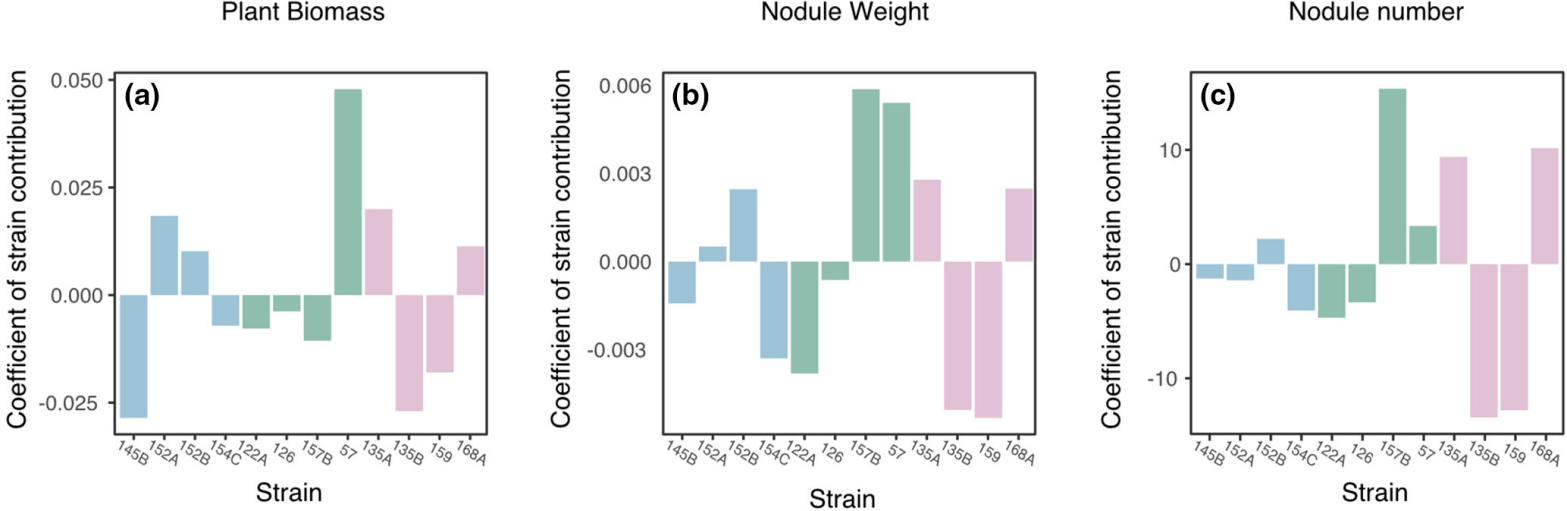
The contribution of individual strains to community function taken from model coefficients for strain effects across the three measured parameters.

The impact of individual genotypes on overall inoculant performance can be deduced from the coefficient for each strain within the model. Analysis of individual genotype contributions revealed several significant contributions of a number of strains to productivity outcomes (Fig. 3). The presence of strain SM57, the highest performing strain in single inoculant pots, within inoculants significantly increased plant biomass across the experiment (Fig. 3a, t=2.660, *P*=0.0088). Secondly, inoculants containing strain SM157B contained higher numbers of nodules (Fig. 3b; t=2.074, *P*=0.040) and also tended to have higher nodule weight though this was not significant (Fig. 3c; *P* >0.01).

**Fig. 4.**
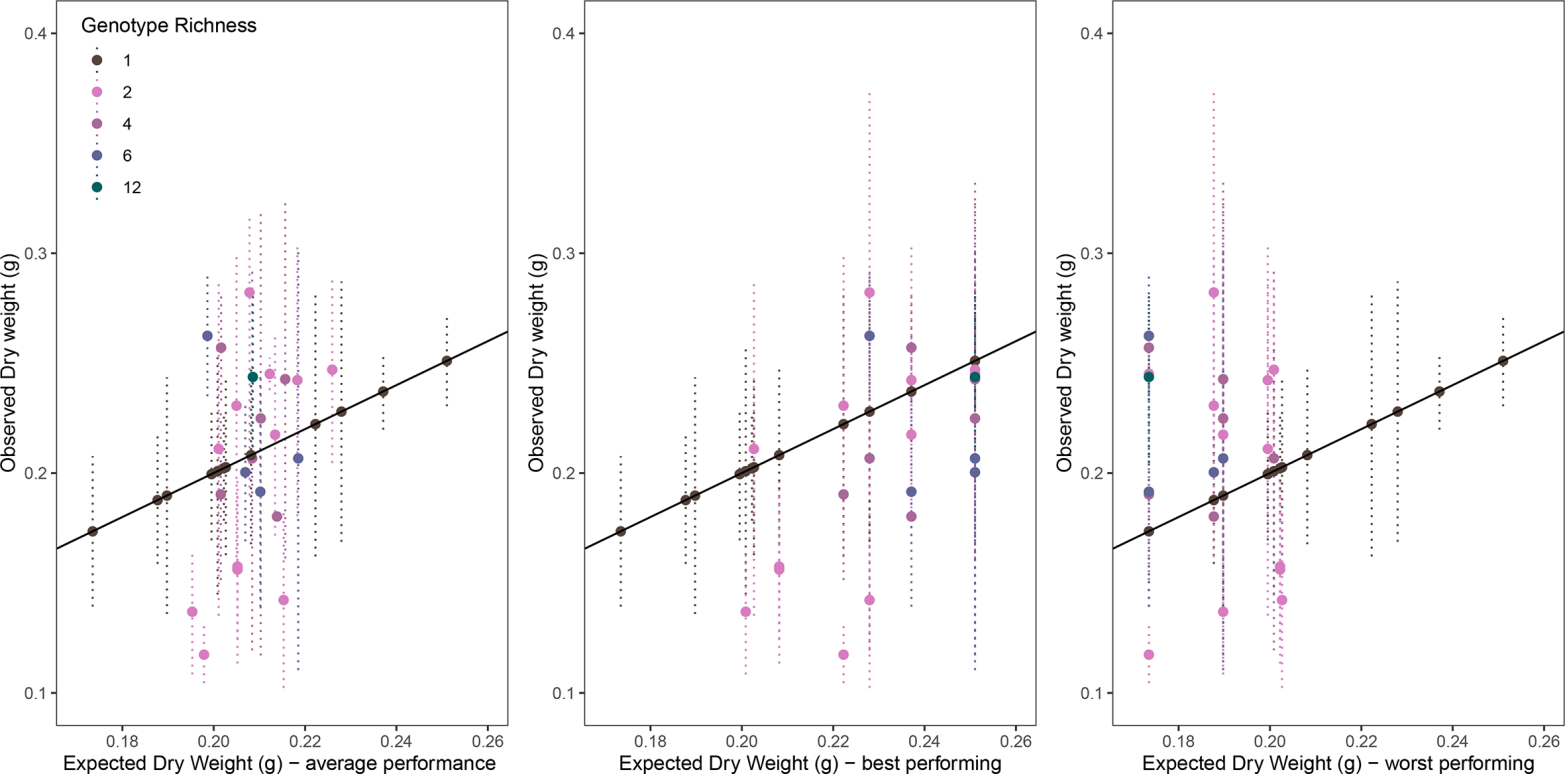
Comparison of observed inoculant productivity, as total plant biomass, with expected productivity calculated from individual isolate inoculants. Expected productivity calculated as (a) the average of all constituent strains, (**b**) the biomass of the most productive strain in the community, and (c) the biomass of the least productive strain in the community.

To further understand the interaction between inoculant make-up and inoculant performance for plant productivity the observed plant biomass of multistrain inoculants was compared to the outcomes that would be predicted based on the growth performance of single inoculants (Fig. 4). Expected values for mixed inoculants were calculated from the plant biomass values associated with each strain as a single strain inoculant in three ways - as the mean, maximum or minimum values for strains in a mixed inoculant. Correlations between observed and expected outcomes were significant for both mean (F_1,34_=13.19, *P*=0.00091, Rsq=0.28) and maximum (F_1,34_=15.78, *P*=0.00035, Rsq=0.317) expected values with a marginally better fit for predicted values based on the highest performing strain (AIC: mean=−137.71, maximum=−139.635). Predicting inoculant outcome based on the least performing strain gave the least predictive power (F_1,34_=3.697, *P*=0.0629, Rsq=0.098, AIC=−129.624).

## Discussion

Rhizobial populations show a high degree of standing genetic diversity. Consequently, hosts engage in symbioses with many genotypes simultaneously. Here we investigated the impact of such intraspecific symbiont diversity on the symbiosis between *Rhizobium leguminosarum* and clover using a biodiversity-ecosystem function framework [19]. This approach allows us to unpick the impact of symbiont diversity, dissecting the impact of diversity itself - i.e. complementarity effects driven by niche partitioning and facilitation - and of strain identity - i.e. selection effects driven by the characteristics of members within a community [16].

We examined the impact of diversity-function relationships within a 12 strain community of *R. leguminosarum *. Estimates of growth responses to key environmental variables - nutrient stress, pH and temperature - for these 12 genotypes showed that strains exhibit significant phenotypic diversity across the three divergent genospecies. However this was not reflected in strain performance within the symbiosis with no detectable effect of genospecies or phenotype on the performance of individual inoculants. Similarly in mixed inoculants no significant effects of diversity were identified, however overall the relationship between productivity and the different measures of diversity tended to be positive, albeit non-significant. This relationship was most pronounced between species richness and symbiotic performance - in contrast to the far weaker effect of genetic diversity and a slightly negative interaction with phenotypic diversity. This lack of significance is likely, in part, due to high variance between replicate blocks within the experiment, which reduced our ability to detect fine scale effects between inoculants. Despite this several lines of evidence point towards strain identity playing a significant role in the diversity effects observed. Within the model significant effects of strain identity were detectable in two instances; mixed inoculants containing strains SM57 and SM157B performed significantly better for symbiotic performance (i.e. plant biomass) and rhizobial productivity (i.e. nodule number/weight) respectively. In both cases, these strains represented the most productive genotype in individual inoculants for each measure. Secondly, across all strains the contribution to mixed inoculant performance (measured as coefficients for strain identity) was positively correlated with the performance of single strains in individual inoculants suggesting a more subtle contribution of strain composition than was detectable in the model. These effects are consistent with the selection model of biodiversity-ecosystem function, whereby greater diversity increases function by increasing the probability of productive strains/species/individuals being present in the community [16]

Relatively few studies have investigated the impact of rhizobial diversity on productivity within the symbiosis. Generally these studies find conflicting impacts of diversity itself, ranging from neutral to negative, but taken together support an important role for strain identity/inoculant composition in the effectiveness of multistrain inoculants. In natural communities, diversity has been associated with reduced host productivity within the rhizobia-*Acacia* symbiosis [12]. Interestingly, diversity was also found to be reduced in association with plants that were better able to sanction their rhizobial symbionts relative to those with better symbiont discrimination [12], which may indicate a preference for reduced diversity by the plant in natural communities which may contain ineffective cheater strains. Inoculant studies, however, generally support the finding of our study. Multistrain inoculants with low levels of diversity (three strains) in various crop species (soya, chickpea and bean) showed equal effectiveness with individual strains [14, 15], and where strain differences were detectable, showed equal effectiveness with the best performing individual strain [15]. This is consistent with our finding that the benefits of multistrain inoculants are driven by selection. A more detailed study, comparing the effectiveness of one, two and four strain inoculants in *Acacia* found that diversity significantly reduced plant productivity although four strain inoculants performed better than two [11] which could potentially indicate a more positive relationship at higher diversity levels. Similar to our study, Barrett *et al*. [11] found that the primary driving factor was strain identity. Thus, overall biodiversity-function relationships appear to be relatively mild for rhizobial symbionts across the scales investigated here, however these studies consistently suggest that strain composition plays a significant role in determining the outcome of rhizobial diversity.

This finding has important implications for the application of rhizobial inoculants in sustainable agriculture. The symbiotic performance of rhizobial strains are highly context dependent, varying both by environment and by host genotype [13]. ‘Elite’ strains selected for use as inoculants are typically tested under controlled conditions that - for clear practical reasons - cannot encompass all field conditions and in particular exclude aspects of competition with the local, and thus locally adapted, microbiome. Consequently, rhizobial inoculants typically contain 1–3 strains very coarsely tailored to a handful of specific farm conditions [3]. Inoculant bacteria frequently fail to establish and/or are outcompeted by less productive native rhizobia [23, 24]. Increasing the diversity of inoculants thus could be beneficial in two non-mutually exclusive ways. Firstly, positive diversity-function relationships could directly increase productivity. Secondly and perhaps more importantly, diverse inoculants could also be used as a bet hedging strategy, increasing the probability of introducing a ‘locally elite’ symbiotic partner for any given environment, i.e. through the selection model of biodiversity-ecosystem function. In our study we find little evidence that significant gains are to be made from diversity alone. However, the performance of multistrain inoculants was best predicted by the performance of the best performing strain, and poorly predicted by the performance of the least productive strain. This suggests that there is little cost to increasing the diversity of inoculants, but potentially much to gain by increasing the probability of introducing a (hard to predict) locally successful strain. A critical test would be to assess the interaction between diversity and environmental variation to see if diverse inoculants are more resilient to environmental heterogeneity than single strain inoculants.

## Supplementary Data

Supplementary material 1
