# Supplementary material for: The impact of intra-specific diversity in the rhizobia-legume symbiosis
Source: Microbiology (Reading). 2021 Apr 8;167(4):001051. doi: 10.1099/mic.0.001051 (PMC8289218; doi:10.1099/mic.0.001051)
Supplement: Supplementary material 1 [file mic-167-1051-s001.pdf]

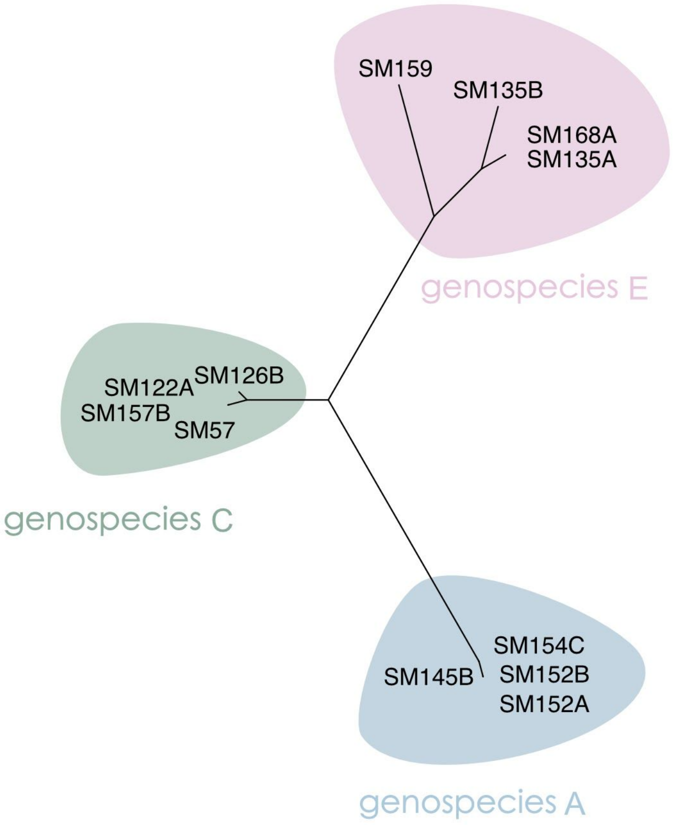

FIgure S1. Phylogenetic relationship between strains showing 3 clades corresponding to genospecies A, C and E. The tree was built using a clustal wallis alignment of housekeeping gene, recA, and assembled using ape4 in R.

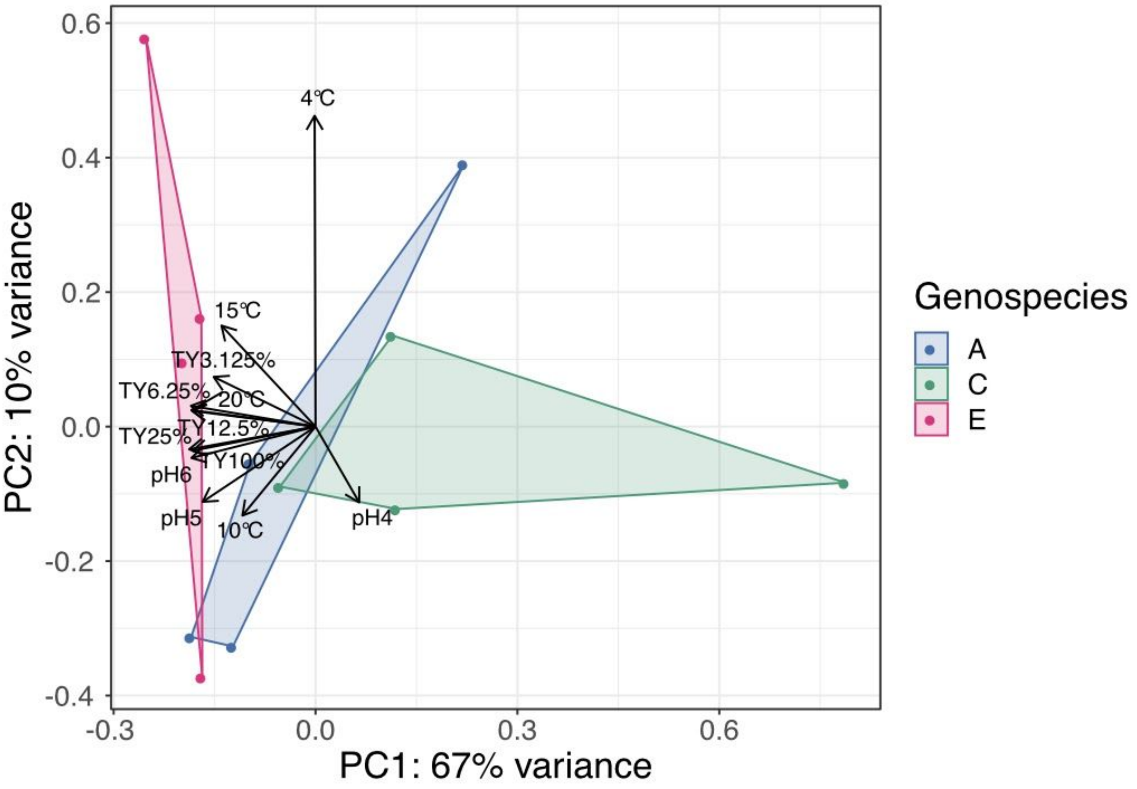

Figure S2. Phenotypic divergence between strains belonging to 3 genospecies. Principal Components Analysis showed large variation in growth phenotypes of strains, with genospecies E strains found to separate from genospecies C. Points represent strains and are coloured by genospecies. Spread of the strains indicates phenotypic variation in ability to grow under different Tryptone Yeast (TY) broth treatments. Vectors show the association of variables conferring growth ability under manipulated TY treatments to the first two principal components.

Model variables in order	df	Plant biomass		Nodule wet weight		Nodule number	
		F	AIC	F	AIC	F	AIC
Block effect	2	52.86*	-338.065	30.37 *	-760.611	10.19*	1376.042
Genotype richness (linear)	1	1.564	-341.642	0.504	-763.122	0.05554	1373.986
Average relatedness	1	0.997	-342.649	0.00053	-763.122	0.08397	1373.901
Phenotypic diversity	1	0.159	-342.811	0.0092	-763.132	0.2657	1373.632
Strain Identity	12	1.056	-336.008	0.629	-751.143	1.164	1379.146

Supplementary Table 1. Model output for each sequential linear model testing for effects of diversity metrics on three measured plant variables. Dependent variables were added to the model in order and each model interrogated for AIC. Asterisks denote significant effects at $p < 0.05$.

		model 1. Genotype first		model 2. Average relatedness first	
		F	R Sq	F	R Sq
Plant Biomass (total dry weight, g)	Genotype richness (linear)	1.564	0.0109	2.098	0.0146
	Average relatedness	0.997	0.00697	0.3212	0.00226
Nodule weight (g)	Genotype richness (linear)	0.504	0.00354	0.384	0.0027
	Average relatedness	0.000531	3.74e-06	0.0699	0.000492
Nodule number	Genotype richness (linear)	0.05554	0.000391	0.0121	8.486e-5
	Average relatedness	0.08397	0.000591	0.137	0.000963

Supplementary Table 2. Model output for each sequential linear model testing for effects of diversity metrics on three measured plant variables. Summary statistics are shown from two versions of each model, model 1 where genotype richness appears first and model 2 where average inoculant diversity appears first.
